# A Case for a Functional Actin Network in the Nucleus

**DOI:** 10.1371/journal.pbio.0020300

**Published:** 2004-08-24

In June, muscular dystrophy patients lost one of their most passionate advocates to a rare form of this degenerative neuromuscular disorder—thirteen-year-old Mattie Stepanek. In his short life, Stepanek wrote five volumes of inspirational poetry, topping the *New York Times* bestseller list and winning accolades from the likes of Jimmy Carter. A wide range of inherited disorders falls under the rubric of muscular dystrophy, but all involve some form of progressive muscle wasting. Stepanek's condition impaired nearly all of his body's functions, but other more common forms, including Emery-Dreifuss muscular dystrophy (EDMD), selectively target skeletal muscle and induce cardiac abnormalities.

EDMD is caused by mutations in either of two genes: one encodes lamin A, a structural protein associated with the nucleus, and the other encodes a nuclear membrane protein called emerin. Lamins, a major component of the structural network that supports the nuclear envelope, help the nuclear envelope maintain structural integrity and absorb mechanical stress without rupturing. (Structures that support the nucleus and regulate molecular traffic between the cytoplasm and nucleus are collectively referred to as the nuclear envelope. They include the inner and outer nuclear membranes, the nuclear pore complexes, and a network of lamin filaments, called the nuclear lamina, near the inner membrane.) Emerin binds to proteins that regulate gene transcription. Emerin and lamins are found in most cell types, yet EDMD attacks only skeletal muscles, major tendons, and the cells that regulate cardiac muscle contraction. So where does this tissue specificity come from?

One theory suggests that emerin selectively targets proteins that specifically regulate gene expression in EDMD-affected tissues. Another theory proposes that emerin provides structural support to the nuclear envelope and that emerin mutations are most destructive in tissues subjected to mechanical stress—like skeletal muscle and tendons. Current evidence supports both models. Recent studies suggest that emerin forms complexes with actin—the mother of all structural proteins. Actin proteins can join together (polymerize) to form a variety of filaments. However, given longstanding doubts that actin exists in the nucleus, let alone functions there, researchers were unsure what the findings might indicate. Now James Holaska, Amy Kowalski, and Katherine Wilson propose that emerin not only functions as a structural protein in the nucleus but that it does so by interacting with actin.[Fig pbio-0020300-g001]

**Figure pbio-0020300-g001:**
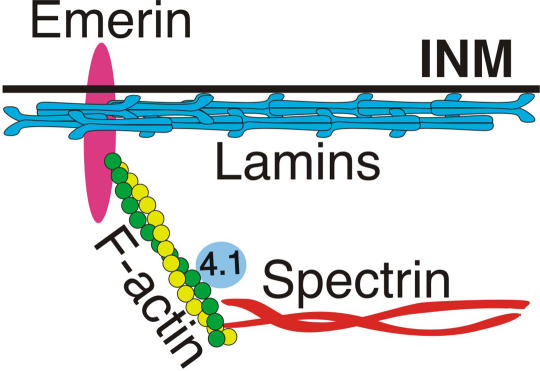
Interactions of structural proteins at the nuclear membrane

Evidence that emerin and lamin A can form multiprotein complexes comes primarily from experiments in test tubes. To get a sense of the physiological significance of these findings, Wilson and colleagues purified emerin-binding proteins from the nuclei of living cells. They found that emerin binds to polymerized actin and, in fact, appears to stimulate polymerization. By binding and “capping” a specific end of the actin filament, emerin prevents filament de-polymerization (disassembly), effectively increasing the rate of actin polymerization by four- to twelve-fold. The authors propose that emerin “promotes the formation of a nuclear actin cortical network,” which could serve to anchor membrane proteins and lamin filaments to the inner nuclear membrane and thus enhance the structural integrity of the nuclear envelope. Whether emerin also interconnects the lamin and actin filament networks at the nuclear envelope—which could significantly reinforce its mechanical strength—will have to await further study.

Muscle contraction places enormous stress on cell membranes. These results suggest that actin-based networks, in addition to lamin networks, support the structural integrity of the nuclear envelope. Defects in proteins involved in either network could compromise nuclear structure, which could in turn disrupt the cell's gene expression program, for example, or rupture the cell membrane, killing the cell. Subtle defects in proteins important for muscle cell integrity can cause several forms of muscular dystrophy. Now it appears that emerin defects could cause EDMD in part by compromising the mechanical integrity of nuclei in muscle cells and tendons.

